# Reconstruction-free positron emission imaging: Fact or fiction?

**DOI:** 10.3389/fnume.2022.936091

**Published:** 2022-07-28

**Authors:** Georg Schramm

**Affiliations:** Division of Nuclear Medicine, Department of Imaging and Pathology, KU Leuven, Leuven, Belgium

**Keywords:** PET, time of flight, image reconstruction, nuclear medicine, molecular imaging

## 1. Introduction

Positron emission tomography (PET) is a quantitative imaging technique based on the detection of pairs of 511 keV photons originating from positron electron annihilation to visualize the spatio-temporal distribution of a radiotracer *in vivo*. Next to X-ray computed tomography (CT) and single photon emission tomography (SPECT), it is one of the classical medical tomographic imaging techniques that allows to derive a three-dimensional distribution of interest (e.g., the activity contration of the radio tracer) based on the measurement of projections of the distribution^1^ which enables non-invasive imaging.

The possibility of deriving a two-dimensional function (distribution of interest) from a set of line integrals (Radon transform) was already discovered by Radon (1). In addition to the original inversion formula provided by Radon himself, many other algorithms have been developed over the years, to derive the distribution of interest from the set of measured projections^2^.

Some of these inversion (or reconstruction) methods are analytic—the most prominent being the filtered backprojection algorithm—while others are iterative (e.g., maximum likelihood or penalized likelihood reconstructions). All analytic and iterative reconstruction methods have in common that (back)projections need to be calculated to derive the distribution of interest. For analytic methods, this is usually a single backprojection, whereas for iterative methods many forward and backprojections need to be calculated. For various reasons, iterative methods, e.g., early-stopped and post-smoothed maximum expectation maximization with ordered subsets (OS-MLEM) (3–5), have replaced analytic reconstruction techniques in PET for many years now which means that a considerable computational effort is needed to reconstruct the image from the measured data.

**Figure 1:**
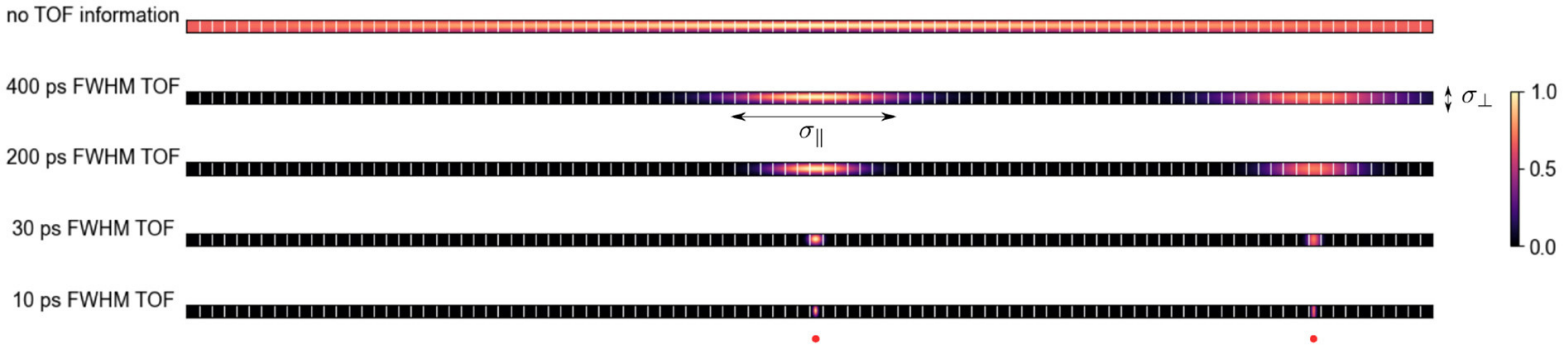
Qualitative probability distribution of the location of two positron-emitting sources (one in the center and one toward the right edge) for a given TOR on PET systems with different TOF resolutions and ca. 5 mm wide crystals. With better TOF resolution the uncertainty along the TOR σ_∥_ decreases. At a TOF resolution of ca. 30 ps FWHM, σ_∥_ is comparable to the uncertainty perpendicular to the TOR σ_⊥_ in a PET system with 5 mm crystals and a radius of 400 mm.

Due to tremendous advances in PET detector technology, the detection of the arrival time difference of the two 511 keV photos with sufficient precision became feasible over the years (6) such that today commercial clinical PET scanners with a coincidence timing resolution of around 200–400 ps full with at half maximum (FWHM) exist. An estimation of the arrival time difference Δ*t* with given uncertainty σ_Δ*t*_ offers additional information which allows to restrict the possible emission location along the tube of response (TOR) into a distribution with spatial uncertainty σ_∥_ along the TOR given by


(1) $$\begin{array}{l} \sigma_{∥}=\frac{c}{2}\sigma_{\Delta t}\;, \end{array}$$

where *c* is the speed of light. As shown in Figure 1, for state-of-the-art time of flight (TOF) PET systems with TOF resolutions between 200 and 400 ps, the uncertainty along the TOR imposed by the TOF information is much bigger compared to the uncertainty perpendicular to the TOR (σ_⊥_) due to the detector width, photon acolinearity, positron range, detector cross-talk and parallax effects.

The benefits of having TOF information for image reconstruction, even in the regime where σ_∥_ ≫ σ_∥_, are numerous and include: improvements in the signal to noise ratio (7), mitigation of limited angle artifacts (8), joint reconstruction of activity and attenuation (9), faster convergence of iterative reconstruction algorithms (10).

In a remarkable work, Kwon et al. (11) recently showed that it is possible to build a PET imaging system based on the detection of Cherenkov photons in two collimated detectors that has a coincidence TOF resolution of 32 ps^3^ meaning that σ_∥_ ≈ σ_⊥_. In their article, the authors argue that by having detectors with such excellent coincidence TOF resolution a PET “image can be directly obtained without any reconstruction step” (**direct positron emission imaging or reconstruction-free PET**).

In this article, we aim to discuss the potential and also the limitations of reconstruction-free PET imaging, considering the physics of the PET data acquisition process and the statistical distribution of the acquired data. Before doing so in the following sections, however, we first need to define what “reconstruction-free” actually means. As mentioned by Kwon et al., we can define reconstruction-free PET imaging as a method that directly generates cross-sectional PET images from the measured TOF PET data after applying a few simple analytic corrections (e.g., using the measured TOF profile along a TOR directly after correction for photon attenuation and detector sensitivities). In other words, reconstruction-free PET should be able to generate cross-sectional PET images without the need to calculate time-consuming (weighted) line or volume integrals and without requiring iterative techniques. Currently, no real full-scale clinical PET systems with “perfect” TOF resolution and adequate photon detection sensitivity exist and it remains to be seen whether such systems will become reality 1 day (6, 12, 13). However, since a roadmap and a challenge toward a 10 ps TOF PET system exist (14), thinking about what the image reconstruction process will look like and how it might differ from the reconstruction process that is being used in current state-of-the-art TOF PET systems is definitely important.

## 2. A statistical perspective on reconstruction-free PET imaging

**Figure 2:**
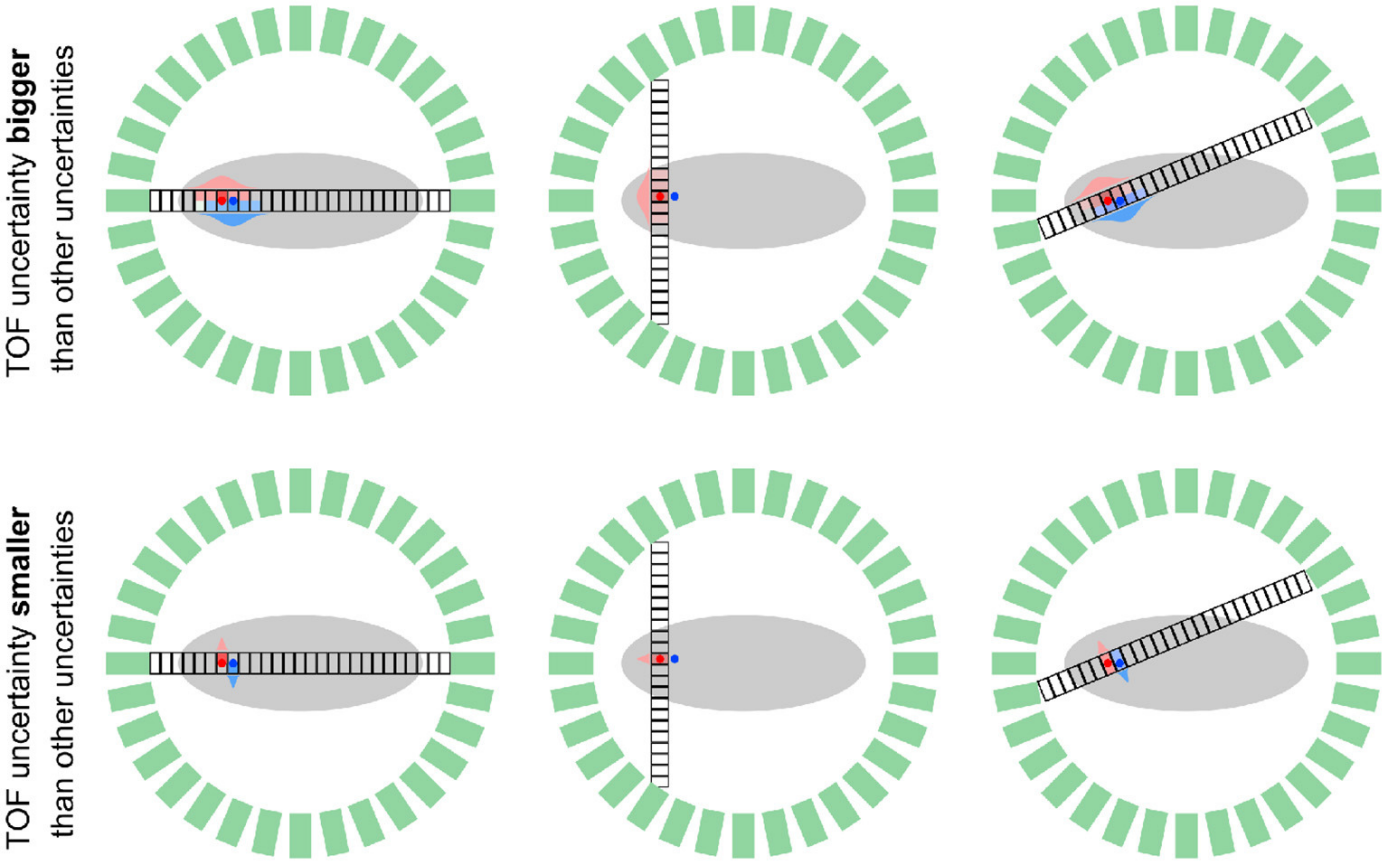
Schematic comparison of a TOF PET system with limited TOF resolution (σ_∥_ > σ_⊥_, top) and “perfect” TOF resolution (σ_∥_ < σ_⊥_, bottom) for two positron-emitting sources (red and blue dots) and three geometrical TORs that are subdivided into “small” TOF bins. In the system with limited TOF resolution, every TOF bin on a fixed TOR is affected by multiple sources as shown by the overlapping red and blue TOF kernels. In contrast, in the system with “perfect” TOF, every TOF bin is only affected by one of the sources, such that a separation of the source locations based on the measured data is possible. Note that in a scanner with realistic detector geometry (multi rings or two bigger parallel plates) every source (voxel) contributes to measured data in multiple data bins (different TOF bins on different geometrical TORs.

In this section, we take a look at the reconstruction problem in PET systems with “perfect” TOF resolution from a statistical perspective. To do so, we consider a PET scanner where the uncertainty along the TOR σ_∥_ due to TOF is much smaller compared to the uncertainty σ_⊥_ caused by finite detector size, detector crosstalk, photon acolinearity and the positron range. Moreover, to simplify the analysis in this section, we assume that the counts in a given TOF bin in the measured data only originate from one voxel in the image to be reconstructed^4^. Note, however, that in realistic scanner geometries, every voxel contributes counts to several bins in the measured data because it is crossed by several TORs as shown in Figure 2. Using this assumption mentioned above, the problem of reconstructing all unknown voxel intensities can be separated into a set of independent problems.

As in current PET systems with limited TOF resolution, the measured data *y*_*i*_ of systems with “perfect” TOF resolution follow a Poisson distribution


(2) $$\begin{array}{l} y_{i}\sim \text{Poisson}\left(c_{ij}\lambda_{j}+{\bar{s}}_{i}\right) \end{array}$$

where *i* is a master index combining the index of the geometrical TOR and the index of the TOF bin along that TOR, λ_*j*_ is the unknown image intensity of a given voxel *j*, *c*_*ij*_ is the system matrix element including the effects of attenuation along the TOR, detection sensitivities and spatial blurring and ${\bar{s}}_{i}$ is the contribution scatter and random contamination with known mean. The reconstruction problem is now to estimate the unknown intensity λ_*j*_ from a set of measured values *y*_*i*_.

One possible approach to estimate λ_*j*_ is to use the maximum likelihood estimator $\lambda_{j}^{ML}$ that can be obtained by maximizing the Poisson loglikelihood that measures the likelihood the measured data *y*_*i*_ given an image estimate ${\hat{\lambda }}_{j}$


(3) $$\begin{array}{l} {\hat{\lambda }}_{j}^{ML}\in \underset{x}{\mathrm{argmax}}\underset{i}{\sum }-\left(c_{ij}\lambda_{j}+{\bar{s}}_{i}\right)+y_{i}\log\left(c_{ij}\lambda_{j}+{\bar{s}}_{i}\right) \end{array}$$

leading to the necessary condition


(4) $$\begin{array}{l} 0=\underset{i}{\sum }c_{ij}\left(\frac{y_{i}}{c_{ij}{\hat{\lambda }}_{j}^{ML}+{\bar{s}}_{i}}-1\right)\;, \end{array}$$

which in the case of no additive contaminations (${\bar{s}}_{i}=0$), has the analytic solution


(5) $$\begin{array}{l} {\hat{\lambda }}_{j}^{ML,{\bar{s}}_{i}=0}=\frac{\sum_{i}y_{i}}{\sum_{i}c_{ij}}\;. \end{array}$$

Unfortunately, if ${\bar{s}}_{i}\neq 0$ and *c*_*ij*_ > 0 for more than three bins in the measured data, (4) has no analytic solution anymore. Therefore, if we want to avoid iterative techniques to estimate λ_*j*_, other estimators for λ_*j*_ have to be used. One possibility for an analytic estimator of λ_*j*_ in the case *s*_*i*_ > 0, is to use the “precorrected” estimator


(6) $$\begin{array}{l} {\hat{\lambda }}_{j}^{PC}=\frac{\sum_{i}y_{i}-s_{i}}{\sum_{i}c_{ij}}\;. \end{array}$$

Another alternative is to use the unweighted least squares estimator


(7) $$\begin{array}{l} {\hat{\lambda }}_{j}^{LS}=\frac{\sum_{i}c_{ij}\left(y_{i}-s_{i}\right)}{\sum_{i}c_{ij}^{2}} \end{array}$$

or the weighted least squares estimator


(8) $$\begin{array}{l} \begin{array}{c} {\hat{\lambda }}_{j}^{WLS}=\frac{\sum_{i}c_{ij}(y_{i}-s_{i})/w_{i}}{\sum_{i}c_{ij}^{2}/w_{i}}. \end{array} \end{array}$$

All of the four estimators ${\hat{\lambda }}_{j}^{ML}$, ${\hat{\lambda }}_{j}^{PC}$, ${\hat{\lambda }}_{j}^{LS}$, and ${\hat{\lambda }}_{j}^{WLS}$ have advantages and disadvantages. The main advantage of ${\hat{\lambda }}_{j}^{PC}$, ${\hat{\lambda }}_{j}^{LS}$, and ${\hat{\lambda }}_{j}^{WLS}$ is that they are unbiased and that they are computationally efficient since analytic expressions for them exist^5^. The main disadvantage of ${\hat{\lambda }}_{j}^{PC}$, ${\hat{\lambda }}_{j}^{LS}$, and ${\hat{\lambda }}_{j}^{WLS}$ is that they are not statistically efficient meaning that asymptotically they do not reach the Cramer-Rao lower bound for their variance (15). In contrast, the maximum likelihood estimator ${\hat{\lambda }}_{j}^{ML}$ is asymptotically consistent (unbiased) and statistically efficient. However, in the presence of additive contaminations ${\bar{s}}_{i}$ and a limited acquisition time (in other words finite number of acquired counts), ${\hat{\lambda }}_{j}^{ML}$ is in general biased. The magnitude of the bias depends on the expected number of the recorded counts $\underset{i}{\sum }c_{i}\lambda_{j}+{\bar{s}}_{i}$ (a lower number of acquired counts leads to more bias) and the ratio between contamination and “true” counts in all data bins ${\bar{s}}_{i}/\left(c_{ij}\lambda_{j}\right)$. Moreover, ${\hat{\lambda }}_{j}^{ML}$ is also less computationally efficient compared to the other estimators since it can be only found with iterative techniques. The choice of the most suitable estimator for λ_*j*_ will depend on

1. The given clinical task and whether low bias or low variance is more important for that task. This trade-off between bias and variance might be e.g., very different for a detection vs. a quantification task.
2. The expectation of the total number of acquired counts $\underset{i}{\sum }c_{ij}\lambda_{j}+{\bar{s}}_{i}$. In a high count regime, ${\hat{\lambda }}_{j}^{ML}$ is probably the optimal estimator since it is statistically consistent and efficient. For a quantification task in the low count regime, one might prefer one of the other estimators because of the bias of ${\hat{\lambda }}_{j}^{ML}$.
3. The available computational resources and time^6^.

We emphasize again that if ${\hat{\lambda }}_{j}^{ML}$ is the preferred estimator—e.g., due to its lower variance—iterative techniques can not be avoided.

## 3. Attenuation correction and estimation of scattered coincidences

As we have seen in the previous section, estimation of the unknown tracer concentration λ_*j*_ in a given voxel *j* in a PET system with “perfect” TOF resolution requires accurate knowledge of the system matrix elements *c*_*ij*_ and the expectation of the additivive contaminations ${\bar{s}}_{i}$. In addition to the effects of intrinsic detection efficiencies and the TOF weight between a given voxel and a TOF bin along a geometrical TOR, the system matrix elements *c*_*ij*_ must include the effect of photon attenuation to obtain PET images with correct regional contrast and absolute quantification. Knowing the 511 keV linear attenuation coefficient μ at every position crossed by a TOR, photon attenuation in PET can be modeled *via* a TOR-dependent factor that can be calculated *via* the linear attenuation law


(9) $$\begin{array}{l} a_{i}=e^{-\int_{\text{TOR}}\mu \left(x\right)dx}\;. \end{array}$$

Therefore, to correctly model the effect of photon attenuation, the calculation of line integrals through the known attenuation image (the forward projection of the attenuation image) is required. Strictly speaking, this means that quantitative PET imaging without calculation of any line integral is not possible. Note that it was shown that TOF PET data include attenuation information themselves and that TOF PET data determine the attenuation sinogram up to a constant (9). However, estimating the attenuation sinogram from its derivatives and estimating the missing constant usually requires more advanced iterative techniques such as MLAA (16) or MLACF (17) including the calculation of many forward and back projections.

In addition to the modeling of photon attenuation, the amount of expected scattered coincidences that contribute to ${\bar{s}}_{i}$ in all data bins need to be estimated. Since the amount of detected scattered coincidences depends on the attenuation as well as the activity image, this estimation is usually done in an iterative way. First, an initial PET image, where the contribution of scattered coincidences is ignored, is estimated. This initial image that overestimates the true activity concentration in conjunction with the attenuation image is then used as input to either an analytic scatter simulation (18, 19) including the calculation of many (weighted) line integrals or as input for dedicated Monte-Carlo simulations to produce a first estimate of the expected amount of scattered coincidences in every data bin. The first scatter estimate can then be used to re-estimate the activity concentration and the updated activity concentration estimate can be used to re-estimate the scatter. This procedure is repeated until a stable estimate for the activity concentration and scatter distribution is reached. Avoiding this iterative procedure was only possible if scattered coincidences could be rejected on the hardware level which is currently not feasible. In theory, detectors with very good energy resolution or directional information on every detected photon would be able to reject scattered coincidences, but reaching that seems at least as challenging as reaching detectors with “perfect” TOF resolution.

## 4. PET imaging requires noise suppression

Since the available acquisition time and the amount of radiotracer that can be safely injected into a patient are limited, the acquired data in PET usually suffer from high levels of Poisson noise that gets transferred into the reconstructed PET image such that techniques for noise suppression are essential to obtain images with practical signal to noise levels. In contrast to PET systems with limited TOF resolution, the noise between neighboring voxels in a system with “perfect” TOF resolution will be uncorrelated, if the reconstruction can be split into independent subproblems for every voxel. To suppress noise in current PET imaging, two main strategies are commonly used. First, the reconstructed image can be post-processed by either applying a conventional smoothing filter or, more recently, by feeding the reconstruction into a pre-trained convolutional neural network (20, 21). Alternatively, instead of using the maximum likelihood or any of the other estimators that describe data fidelity, a maximum a posteriori estimate (MAP) estimator can be used. This is possible by augmenting the Poisson loglikelihood with a term that reflects prior knowledge on the image to be reconstructed (e.g., smoothing priors penalizing a given norm of finite forward differences between neighboring voxels (22, 23). In general, optimizing the augmented MAP objective function is only possible using iterative methods. Recently, it was also shown (24–26) that the combination of iterative reconstruction and trainable convolutional neural networks into unrolled networks can be used to improve the quality of PET images beyond what is possible with classical methods.

## 5. Exploiting the resolution benefit of ultra-fast TOF scanners

For state-of-the-art PET systems where σ_∥_ ≫ σ_⊥_, the fundamental limit for the spatial resolution that can be achieved is dominated by the detector size, photon acolinearity, scanner radius and the positron range as described in Moses (27) meaning that for those systems, the TOF information has no beneficial impact on the spatial resolution. Interestingly, based on Monte Carlo simulations of a ring-like ultra-fast TOF PET system with very thin detectors^7^, Toussaint et al. (28, 29) recently found evidence that in the regime where σ_∥_ ≤ σ_⊥_, TOF information enables PET imaging with resolutions below the fundamental limit imposed by the detector size, photon acolinearity, and the positron range. In their work, Toussaint et al. used the iterative TOF-MLEM algorithm to reconstruct the simulated data and could show that the resolution in the TOF-MLEM reconstructions lead to better resolutions when iterating long as also shown in Gong et al. (30). To mitigate effects that lead to spatial blurring in the reconstructed images, those effects (σ_⊥_, σ_∥_) should be modeled as accurately as possible in the reconstruction process. Since image deblurring in general is an ill-posed and ill-conditioned inverse problem, regularization and iterative methods will be required for finding a stable solution.

## 6. Discussion

All in all, we can say that improving the TOF resolution to a level where the TOF-induced uncertainty parallel to the TOR is smaller than the uncertainty perpendicular to the TOR, will definitely change the way that PET images are estimated from the measured data. In many aspects, the availability of “perfect” TOF resolution will simplify this non-trivial problem. However, as discussed in the previous sections, quantitative PET imaging without calculating line integrals to include the effect of photon attenuation, or without using iterative techniques to e.g., estimate the contribution scattered coincidenes or to find the maximum likelihood or maximum a posteriori estimator, is not feasible. As demonstrated in the proof-of-concept by Kwon et al. (11), in the absence of scattered coincidences, direct PET imaging is in principle possible^8^. However, the question remains whether simple direct (reconstruction-free) methods will yield “optimal” image quality concerning image resolution and noise compared to more advanced (iterative) reconstruction techniques in realistic PET systems with “perfect” TOF resolution. The road toward a clinical system with “perfect” TOF resolution is still long and certainly full of expected and unexpected challenges in the image formation process. For any future PET system with better and better TOF resolution that will be developed along this road, experts in the fields of PET hardware design, modeling of PET acquisition physics, estimation theory and inverse problems, machine learning and image reconstruction have to closely collaborate to ensure that the diagnostic information captured in the acquired data is used in the best possible way. Based on the statistical distribution of the acquired data and the physics behind the acquisition process, it is possible that this process might include a step that deserves the name “reconstruction.”

## Author contributions

The author confirms being the sole contributor of this work and has approved it for publication.

## Funding

This work was supported in part by the NIH grant 1P41EB017183-01A1.

## Conflict of interest

The author declares that the research was conducted in the absence of any commercial or financial relationships that could be construed as a potential conflict of interest.

## Publisher's note

All claims expressed in this article are solely those of the authors and do not necessarily represent those of their affiliated organizations, or those of the publisher, the editors and the reviewers. Any product that may be evaluated in this article, or claim that may be made by its manufacturer, is not guaranteed or endorsed by the publisher.

## Footnotes

^1^In PET, we use the general term “projection” for (weighted) line or volume integrals along a line (LOR) or tube of response (TOR) connecting two photon detectors.

^2^Moreover, inversion methods to invert the more general three-dimensional X-ray transform were developed (2).

^3^Note that the presented system was a proof-of-concept prototype that used two collimated detectors such that the sensitivity of this proof-of-concept system was very low.

^4^This assumption does in general not hold for reconstructions with voxel sizes that are smaller than σ_⊥_ or for ring-like PET scanners where TORs that do not align with the voxel grid exist.

^5^The “optimal” weights *w*_*i*_ that minimize the variance of ${\hat{\lambda }}_{j}^{WLS}$ are in general unknown. A pragmatic choice that tries to resemble the unknown true variances of *y*_*i*_ might be $\text{min}\left(c_{i}{\hat{\lambda }}_{j}^{LS}+{\bar{s}}_{i},{\bar{s}}_{i}\right)$.

^6^Note that the computational effort and time required to find ${\hat{\lambda }}_{j}^{ML}$ for a system with “perfect” TOF resolution will be much smaller compared to systems with “imperfect” TOF resolution since the reconstruction problem can be split into independent 1D subproblems.

^7^Thin detectors were simulated to exclude the effect of depth of interaction leading to parallax errors.

^8^Note that the directly acquired data was also corrected for photon attenuation by calculating the forward projection of the attenuation image.
